# *In vitro* additive interaction between ketoconazole and antimony against intramacrophage *Leishmania (Leishmania) amazonensis* amastigotes

**DOI:** 10.1371/journal.pone.0180530

**Published:** 2017-06-29

**Authors:** Débora Cristina de Oliveira Nunes, Luiz Borges Bispo-da-Silva, Danielle Reis Napolitano, Mônica Soares Costa, Márcia Moura Nunes Rocha Figueira, Renata Santos Rodrigues, Veridiana de Melo Rodrigues, Kelly Aparecida Geraldo Yoneyama

**Affiliations:** 1 Instituto de Genética e Bioquímica, Universidade Federal de Uberlândia, Uberlândia, Minas Gerais, Brazil; 2 Instituto Nacional de Ciência e Tecnologia em Nano-Biofarmacêutica (N-Biofar), Belo Horizonte, Minas Gerais, Brazil; 3 Área de Ciências Fisiológicas, Instituto de Ciências Biomédicas, Universidade Federal de Uberlândia, Uberlândia, Minas Gerais, Brazil; Universidade Federal do Rio de Janeiro, BRAZIL

## Abstract

Leishmaniasis is a group of diseases caused by protozoa of *Leishmania* genus. The currently available treatments for this disease are expensive, present high toxicity and are associated to difficulties of healing and parasite resistance. Therefore, the development of strategies for leishmaniasis treatment is indispensable and includes reposition of existing drugs, as well as drug combination therapy. The aim of this study was to assess the nature of ketoconazole and antimony association on the cytotoxic effect against *Leishmania (Leishmania) amazonensis* amastigotes. The calculated mean sum of fractional 50% inhibitory concentration ($\bar{x}$ΣFIC_50_) was 2.54 and 1.43 for free and intracellular amastigotes, respectively, values that suggest an additive interaction between ketoconazole and antimony concerning to *Leishmania* toxicity only in the intramacrophage parasite form. Despite the clinical efficacy of ketoconazole-antimony combination has been shown in the literature, our study is the first to describe the nature of ketoconazole-antimony interaction against *L*. *(L*.*) amazonensis* amastigotes. Moreover, our results point out the need for future *in vivo* studies to confirm the nature of ketoconazole-antimony interaction and also to determine possible effective dosage regimens related to ketoconazole administration in association with the optimal lower dose of antimony.

## Introduction

Parasitic protozoan diseases are commonly found in the poorest countries of the world and constitute one of the most widely spread human health problem [1]. These diseases are still neglected and unfortunately have received little attention from the pharmaceutical industry and scientific funding agencies [2]. Despite several therapeutic options are available to treat different forms of leishmaniasis, the current chemotherapy of cutaneous leishmaniasis usually relies on antimony-based drugs. However, these drugs produce many side effects and their toxicity associated to the drug-resistant strains have limited therapeutic uses of antimony-based agents [3].

Nowadays, the repositioning of agents already used clinically for other purposes represents an important therapeutic strategy to treat leishmaniasis. In this way, azole compounds, known to inhibit fungal sterol biosynthesis, have been an option described in the literature [4]. Another therapeutic strategy is the combination of drugs with different mechanisms of action. This approach has many therapeutic advantages, such as: increased therapeutic efficacy; decreased toxicity due to reduction in the effective dose; reduced development of drug resistance; and selective synergism against parasite target versus host [3,5,6]. In this respect, the association of antimony-based agents with ketoconazole has been successfully used in human against old world leishmaniasis [7]; however, nothing is known about the pharmacological nature of this association, as well as of its efficacy on new world *Leishmania* species, such as *L*. *(L*.*) amazonensis*. Therefore, in the present study we aimed at investigating the *in vitro* antiparasitic efficacy and the pharmacological nature of antimony associated to ketoconazole against *L*. *(L*.*) amazonensis* amastigotes.

## Materials and methods

### Chemicals

Dimethylsulfoxide (DMSO), Giemsa stain modified solution, 4-(2-hydroxyethyl)-1- piperazineethanesulfonic acid (HEPES), ketoconazole, 3-(4,5-dimethylthiazol-2-yl)-2,5-diphenyl tetrazolium bromide (MTT), potassium antimonyl tartrate trihydrate, RPMI 1640 medium, sodium dodecyl sulphate (SDS), penicillin and streptomycin were purchased from Sigma Chemical Co. (St Louis, USA). Heat-inactivated fetal bovine serum (FBS) (Cultilab, Brazil), Schneider’s insect medium (LGC Biotecnologia, Brazil). All other reagents were analytical grade or superior. Ketoconazole was dissolved in a 1 M stock of dimethyl sulfoxide (DMSO) and stored at -20°C. For experiments, new dilutions were prepared in culture medium to ensure that the DMSO concentration in culture medium did not exceed 0.1%.

### Animals

The experiments were performed using female BALB/c mice supplied by the animal facility of the Federal University of Uberlândia (CBEA/UFU). The animals were housed in a room at 25°C with 12 h light/dark cycles and were provided free access to water and standard chow *ad libitum*. All the protocols used were reviewed and approved by the Ethics Committee on Animal Use of the Federal University of Uberlândia (CEUA/UFU; process n° 36/2013).

### Parasites—Promastigote and free amastigote

*Leishmania (Leishmania) amazonensis* (IFLA/BR/67/PH8 strain) promastigotes were cultured in Schneider’s insect medium, pH 7.0, supplemented with 10% FBS, penicillin (100 UI.mL^−1^) and streptomycin (100 μg.mL^−1^) at 23 ± 0.5°C. Promastigotes used in all experiments were isolated from the stationary growth phase.

Free amastigotes were obtained from footpad of BALB/c mice (6–8 weeks old) previously infected with promastigote forms (1×10^7^ cells/footpad) for 5 to 6 weeks [8]. These parasites were cultured in complete Schneider’s insect medium, at 32 ± 0.5°C, ensuring that these parasites remained as axenic amastigote forms [9], and used within two days.

### Murine macrophage culture

Murine macrophage cell line RAW264.7 was obtained from Rio de Janeiro Bank Cell and cultured in RPMI 1640 medium, supplemented with 5% FBS, penicillin (100 UI.mL^−1^), streptomycin (100 μg.mL^−1^) in 75-cm^2^ flasks. All cell cultures were done at 37°C in humidified air with 5% CO_2_.

### Viability—Macrophage and free amastigote assays

Viability assays in presence of drugs (antimony and/or ketoconazole) were carried on free amastigote forms and murine macrophages cell line RAW264.7, by colorimetric method based on mitochondrial oxidation of MTT reagent (5 mg.mL^−1^, 100 μg/well) [10]. Free amastigotes (5x10^5^ cells/well) and macrophages (2x10^5^ cells/well) were incubated with its respective medium alone (control) or containing ketoconazole (10^−10^ to 10^−3^ M) or antimony (10^−12^ to 10^−3^ M) for 48 hours. The 50% inhibitory concentration (IC_50_) of drugs on cell viability were then determined in each individual experiment considering control values as 100% of viability. This assay was carried out in triplicate and three independent experiments were performed. Cytotoxicity in RAW macrophages and activity against free amastigotes for ketoconazole and antimony were compared using the selectivity index (SI; ratio: IC_50_ RAW macrophage/ IC_50_ parasite).

### Infectivity—Intracellular amastigote assay

Macrophages (4×10^5^) were placed in 24-well plates containing 13-mm diameter glass coverslips. Then, macrophages were infected with *L*. *(L*.*) amazonensis* free amastigotes isolated immediately before their use at 2:1 ratio (free amastigotes/macrophage). After 1h30, plates were washed with PBS to remove non-internalized amastigotes and RPMI medium alone (control) or containing drugs (13.125 to 420 μM for ketoconazole and 0.156 to 5 μM for antimony, both double serial dilutions) were added. Experiments were conducted at 37°C in a 5% CO_2_ humidified incubator for 48 h. Cells on coverslips were fixed and stained with Giemsa stain modified solution for evaluation of the infection index. Infectivity index was defined as the average number of intracellular amastigotes in infected macrophages multiplied by percentage of infected macrophage; 100 cells per coverslip were blind counted. The IC_50_ values of drugs on the infectivity were then determined in each individual experiment considering control values as 100% of infectivity. This assay was carried out in triplicate and two independent experiments were performed.

### Isobologram

The interaction between ketoconazole and antimony was *in vitro* evaluated by using the modified isobologram method according to [5, 11], with modification. Briefly, to construct an isobologram and determine the type of interaction between drugs, the prerequisite is to know the potency and a form of dose-effect curve of each drug alone. The IC50 values obtained in the experiments described in ‘Viability—macrophage and free amastigote assays’ and ‘Infectivity—Intracellular amastigote assay’ subsections were used to determine the maximum concentration of each drug in the combination assay ensuring that IC50 was in the midpoint of curves, except for the intracellular amastigote assay; in this case, the IC_50_ falls on the third point (from the maximum concentration used), that was not the midpoint, since highest concentrations of both drugs necessary to complete the curves were toxic for macrophages. The highest concentration of the solutions were prepared in proportions of 5:0, 4:1, 3:2, 2:3, 1:4 and 0:5 of ketoconazole and antimony, which were serially diluted to the ninth or sixth well of microplate for free amastigote or intracellular amastigote assays, respectively; this assay was carried out in duplicate (infectivity assay) or triplicate (viability assay) and two independent experiments were performed. Assays of combinations in each ratio allowed getting concentration-effect curves and therefore the IC_50_ for each combination ratio in relation to ketoconazole and antimony. Then, fractional inhibitory concentrations (FICs) at the IC_50_ level (FIC_50_) were calculated for both drugs, as follows: FIC50 = IC50 drugs in combination/IC50 drug alone. FIC_50_s of each drug ratio (i.e, 4:1, 3:2, 2:3 and 1:4) were used to build isobolograms. It is important to mention that isobolograms constructed using FIC values are also known as normalized isobolograms [6]. Moreover, the sum of the FIC_50_s for each ratio, also referred as combination index [6], was determined (ΣFIC_50_s = FIC_50_ ketoconazole + FIC_50_ antimony), and an overall mean of ΣFIC_50_s ($\bar{x}$ΣFIC50) was calculated. Finally, the average of the ΣFIC50 was used to classify the nature of interaction: ‘Synergy’ defined as mean ΣFIC ≤ 0.5; ‘Indifference/Additive’ as mean ΣFIC > 0.5 and ≤ 2; and ‘Antagonism’ as mean ΣFIC > 2 [12].

### Statistical analysis

In the experiments, the 50% viability/infectivity inhibition values (IC_50_) were estimated by fitting the concentration-response data to the four parameter logistic equation through nonlinear regression analysis, according to the following equation: as follows: Y = Bottom + (Top-Bottom)/(1+10^((LogEC50-X)*HillSlope)) [13]. Moreover, IC_50_, FIC_50_, ΣFIC_50_s and $\bar{x}$ΣFIC_50_ were calculated for each individual experiment using GraphPad Prism 5.0 (GraphPad Software Inc., San Diego, USA), and results were expressed as the mean and standard error of at least two independent experiments.

## Results

### In vitro susceptibility of amastigotes and macrophages to individual drugs

**Fig 1:**
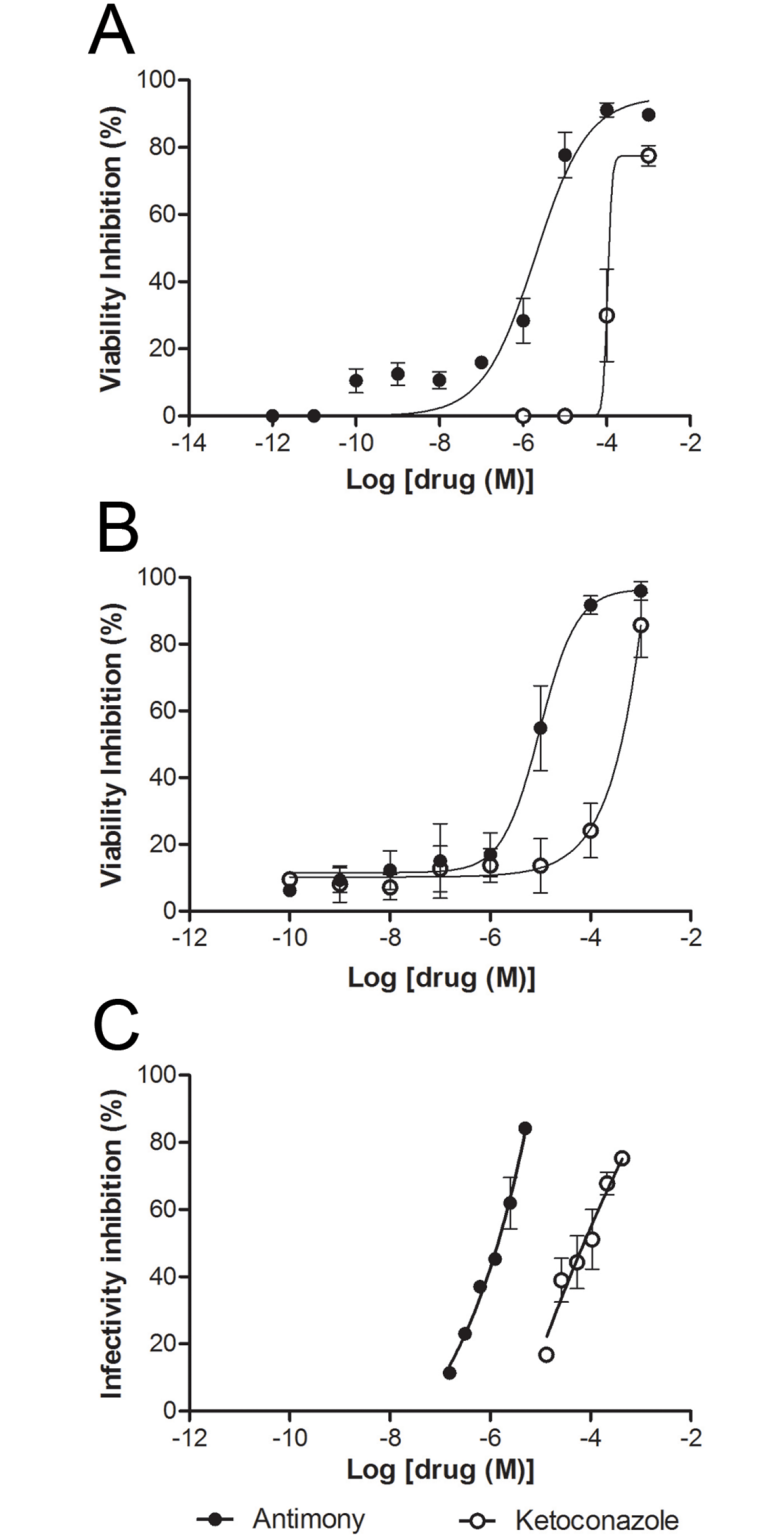
Cytotoxic effects of isolated drugs (ketoconazole and antimony) on free amastigotes, murine macrophages cell line RAW264.7 and intracellular amastigotes. Concentration-effect curves to ketoconazole (white points) and antimony (black points) against *L*. *(L*.*) amazonensis* free amastigotes **(A),** murine macrophages **(B)** or intracellular amastigotes **(C)**. Data represent mean ± SEM of two to three independent experiments performed in triplicate.

Both drugs caused a concentration-dependent inhibition on amastigote viability after 48 hours of incubation presenting IC50 values of 105.5 μM for ketoconazole and 2.5 μM for antimony (Fig 1A). Concentration-dependent inhibition on macrophages cell line RAW viability was also observed (Fig 1B). Ketoconazole was much less toxic to macrophage than antimony (Fig 1B; IC_50_ = 436.5 vs. 7.3 μM; ketoconazole vs. antimony) presenting selectivity index of approximately 4.1 and 2.9 for ketoconazole and antimony, respectively. Both drugs also inhibited intracellular amastigotes in a concentration-dependent way, presenting IC_50_ of 75 and 1.5 μM for ketoconazole and antimony, respectively (Fig 1C).

### Effect of ketoconazole and antimony combination on amastigotes and isobologram

**Fig 2:**
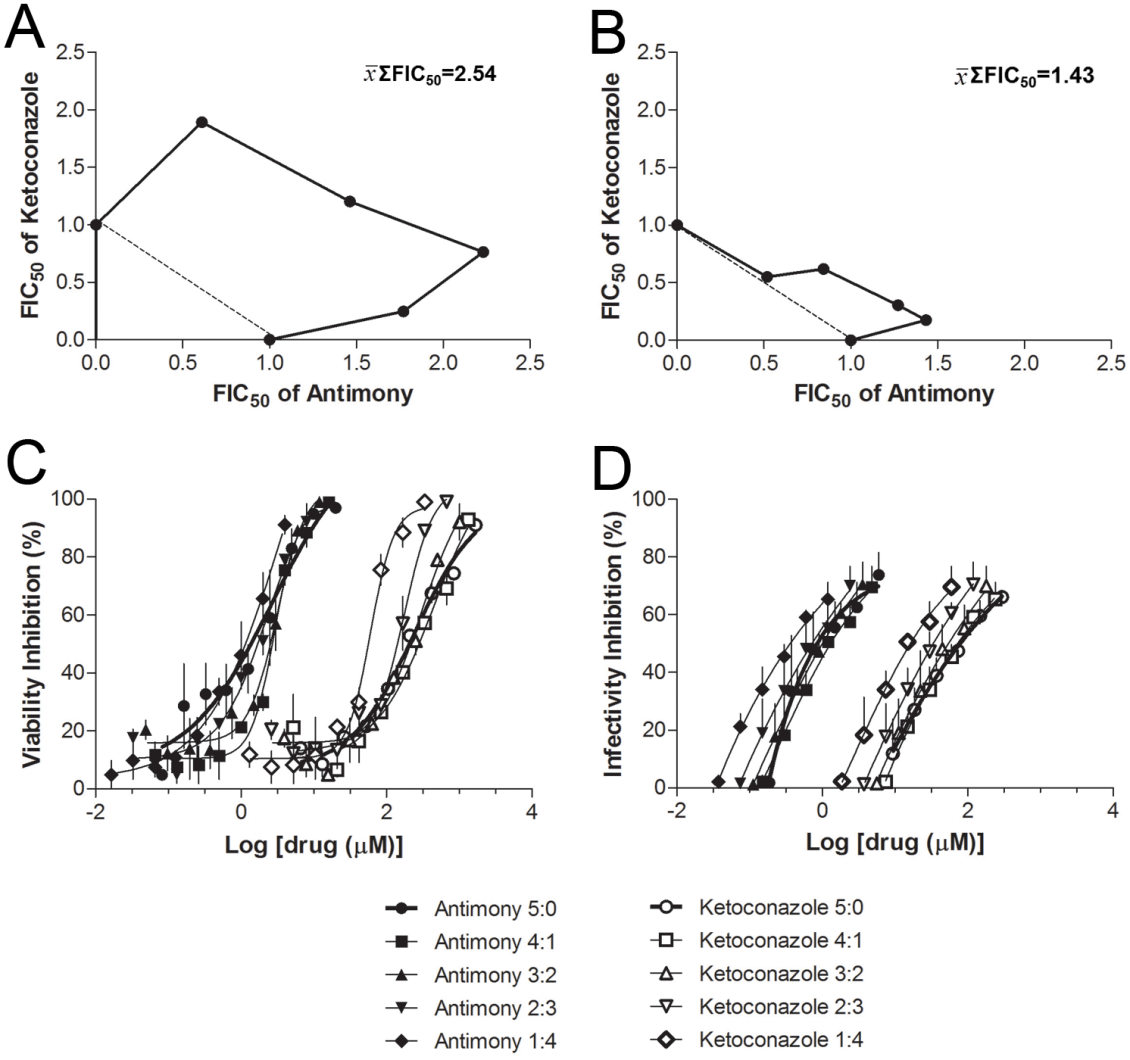
Ketoconazole and antimony combination on *L*. *(L*.*) amazonensis* amastigotes. Isobologram representing *in vitro* ketoconazole-antimony interaction against *L*. *(L*.*) amazonensis* free **(A)** or intracellular **(B)** amastigotes by a fixed-ratio method based on the IC50. Dashed line represents an ideal theoretical line for the additive effect; the $\bar{x}$ΣFIC50 for all interactions tested is also shown. Concentration-effect curves concerning the inhibition of *L*. *(L*.*) amazonensis* free **(C)** and intracellular **(D)** amastigotes by different combinations of antimony (black points) and ketoconazole (white points). Data represent mean ± SEM of two independent experiments performed in triplicate.

This experimental approach allowed the determination of FIC50 values for each combination. Overall mean of ΣFIC50 for free amastigotes assay ranged from 2.01 to 2.99 and the $\bar{x}$ΣFIC50 was 2.54 ± 1.7. For intracellular amastigotes, the overall mean of ΣFIC50 ranged from 1.07 to 1.60 and the $\bar{x}$ΣFIC50 was 1.43 ± 0.38. The IC50, FIC50, ΣFIC50 and $\bar{x}$ΣFIC50 values are given in Table 1 and the corresponding isobolograms are shown in Fig 2A and 2B. The sigmoidal concentration-effect curves for each combination are present in Fig 2C and 2D.

**Table 1:** IC_50_, FIC_50_ and ΣFIC_50_ of antimony-ketoconazole combination against *L*. *(L*.*) amazonensis* free and intracellular amastigotes.

Assay	Combination Rate		Combined drugs					
			IC_50_		FIC_50_		Σ FIC_50_	$\bar{x}$ΣFIC_50_
	Antimony	Ketoconazole	Antimony	Ketoconazole	Antimony	Ketoconazole		
**Free amastigotes**	0	5	---	254.80 ± 60.20	---	---	---	2.54±1.23
	1	4	0.97 ± 0.03	432.75 ± 94.05	0.61 ± 0.31	1.89 ± 0.81	2.50 ± 1.13	
	2	3	2.29 ± 0.02	290.15 ± 2.95	1.46 ± 0.78	1.20 ± 0.27	2.66 ± 1.05	
	3	2	3.13 ± 0.64	175.40 ± 36.00	2.23 ± 1.50	0.76 ± 0.32	2.99 ± 1.82	
	4	1	2.87 ± 0.20	60.38 ± 4.33	1.77 ± 0.87	0.24 ± 0.04	2.01 ± 0.91	
	5	0	2.22 ± 1.20	---	---	---	---	
**Intracellular amastigotes**	0	5	---	89.93 ± 14.77	---	---	---	1.42±0.27
	1	4	0.37 ± 0.07	51.73 ± 21.54	0.52 ± 0.31	0.55 ± 0.14	1,07 ± 0.16	
	2	3	0.88 ± 0.50	62.04 ± 47.59	0.84 ± 0.21	0.62 ± 0.42	1.46 ± 0.21	
	3	2	0.98 ± 0.27	29.66 ± 17.64	1.27 ± 0.69	0.30 ± 0.14	1.57 ± 0.55	
	4	1	1.20 ± 0.44	17.15 ± 10.39	1.43 ± 0.67	0.17 ± 0.08	1.60 ± 0.59	
	5	0	1.27 ± 0.91	---	—	---	---	

Data expressed as mean ± SEM.

## Discussion

Ketoconazole- and antimony-induced *Leishmania* toxicity appears to be related to the inhibitory effects of these drugs on different parasite metabolic pathways [4, 14]. Therefore, in the present study we tested the hypothesis that the combination of ketoconazole with antimony could improve the antileishmanial effect of these agents. It has been shown that Sb^5+^ should be reduced by the host cells to Sb^3+^, the active form against parasites [15, 16]. Thus, since both intramacrophage or free parasites were analyzed, and to avoid biases due to variability in macrophage drug conversion, antimonyl tartrate (Sb^3+^) was used instead meglumine antimoniate (Sb^5+^). Despite ketoconazole was less potent than antimony in inducing *L*. *(L*.*) amazonensis* free (*ca*. 40 times) and intracellular (*ca*. 50 times) amastigotes death *in vitro*, ketoconazole was much less toxic to macrophages than antimony. These data strongly suggest that this azole compound may be used against parasite-infected macrophages ensuring safety and efficacy.

Previous clinical trials studies showed the efficacy of ketoconazole alone in several *Leishmania* species around the world [17, 18, 19, 20], and the literature also pointed out that ketoconazole efficacy is comparable to those of antimonial based drugs [21, 22, 23]. However, to our knowledge, only one study focused on the combined therapy of ketoconazole and antimony. In that study it was observed that sodium stibogluconate (SSG) combined with ketoconazole was more effective than SSG alone to treat human cutaneous leishmaniotic lesions [7]. It is important to mention that the clinical study cited above was conducted in an Asian country where the common etiologic agents for cutaneous leishmaniasis are *L*. *major*, *L*. *tropica*, and *L*. *aethiopica*; however, in our study we focused on *L*. *(L*.*) amazonensis*, one of the typical species related to the disease in the America continent [1].

FIC is a widespread index used to study drug interactions and many critical values have been arbitrarily used to define the nature of drug combination [6, 24, 25, 26, 27]. The additivity range of 0.5 to 4.0 has been recommended by some expertise in the field [26] and employed in several studies concerning the interactions of antileishmanial drugs [11, 28, 29, 30]. However, based on the absence of *in vitro-in vivo* correlation studies, concerns about the validity of this arbitrarily chosen FIC were raised, and a more symmetrical additivity range of 0.5 to 2.0 was suggested [12]. In the present study, two different experimental conditions were perforned, including the amastigote-macrophage model, the gold standard assay for determining the intrinsic drug susceptibility of intracellular amastigotes [31]. According to the more symmetrical additivity range mentioned above, the $\bar{x}$ΣFIC (1.42) observed in our study indicate an additive nature to ketoconazole-antimony combination against intramacrophage amastigote. Moreover, the ketoconazole-antimony combination resulted in ΣFICmin and ΣFICmax of 1.07 and 1.60, respectively, values that are within the range observed in self-drug additive combination studies [12]. On the other hand, the $\bar{x}$ΣFIC (2.54) for free amastigotes suggests an antagonic interaction between ketoconazole and antimony. Despite we have no explanation for this discrepancy these results suggest altogether that the intracellular environment alter the nature of the interaction between ketoconazole and antimony against *L*. *(L*.*) amazonensis*. It is noteworthy that although experiments carried out with promastigotes or axenic amastigotes are easier to perform, to use intracellular amastigotes is clinically more relevant once this parasite form bears a closer similarity to the *in vivo* situation and correlate better with the treatment outcome [32, 33]. The use of lower doses of antimony associated with ketoconazole has the advantage of reducing side effects and limits the development of resistant strains, an emerging worldwide therapeutic problem. Therefore, considering the toxicity of antimony, its indifferent interaction with ketoconazole against intramacrophage amastigote could be useful and should be confirmed *in vivo*.

Moreover, cutaneous lesion may become susceptible to colonization with a number of microorganisms, such as pathogenic or opportunistic fungi that could cause secondary infections [34]. Hence, beyond the suggestion (based on our *in vitro* data) that direct effects of the ketoconazole in combination with antimony on *Leishmania* death could occur *in vivo*, ketoconazole could also improve the leishmaniotic lesion due to its action on fungi.

It is important to mention that we analyzed the interaction just in relation to IC50 level, since we obtained inhibition percentages nearby but less than 100% for some combination within the dilution range used; this fact preclude the determination of all the FIC90 necessary for the data analysis. In attempt to reach 100% of inhibition we increased the ketoconazole concentration, however, in this condition the drug precipitated. It is important to mention that analyzing data using only IC50 level do not invalidate our results and interpretations, since the evaluation of FIC at IC90 levels serve only as rebuttal [35, 36].

In conclusion, although the clinical efficacy of ketoconazole-antimony combination has been shown in the literature, our study is the first to describe the nature of ketoconazole-antimony interaction against *L*. *(L*.*) amazonensis* both in free and intracellular amastigotes. The additive interaction of ketoconazole-antimony combination in the intramacrophage parasite form points out that future *in vivo* experiment should be conducted to confirm this interaction and also to determine possible effective dosage regimens related to ketoconazole administration in association with the optimal lower dose of antimony.
